# Supplementary material for: High bycatch rates of manta and devil rays in the “small-scale” artisanal fisheries of Sri Lanka
Source: PeerJ. 2021 Sep 8;9:e11994. doi: 10.7717/peerj.11994 (PMC8434810; doi:10.7717/peerj.11994)
Supplement: Supplemental Information 2 — HK = Hong Kong; LK = Sri Lanka; T = commercial trade; W = Wild. [file peerj-09-11994-s002.docx]

**Table S1:** Reported trade by Sri Lanka (CITES Trade Database, 2021) of CITES Appendix II listed mobulid rays between 2010 and 2020. *HK = Hong Kong; LK = Sri Lanka; T = commercial trade; W = Wild*.

| **Year** | **Taxon** | **Importer** | **Exporter** | **Exporter reported quantity** | **Unit^‡^** | **Term** | **Purpose** | **Source** |
| --- | --- | --- | --- | --- | --- | --- | --- | --- |
| 2015 | *Manta* spp. | HK | LK | 1000 | kg | derivatives | T | W |
| 2018 | *Mobula japanica* | HK | LK | 550 | kg | gill plates | T | W |
| 2018 | *Mobula japanica* | HK | LK | 550 |  | gill plates | T | W |
| 2018 | *Mobula japanica* | HK | LK | 225 | kg | live^*^ | T | W |
| 2018 | *Mobula tarapacana* | HK | LK | 450 | kg | gill plates | T | W |
| 2018 | *Mobula tarapacana* | HK | LK | 300 |  | gill plates | T | W |
| 2018 | *Mobula tarapacana* | HK | LK | 200 | kg | live^*^ | T | W |
| 2019 | *Manta birostris* | HK | LK | 750 | kg | gill plates | T | W |
| 2019 | *Mobula* spp. | HK | LK | 1050 | kg | gill plates | T | W |
| 2019 | *Mobula japanica* | HK | LK | 630 | kg | fins^†^ | T | W |
| 2019 | *Mobula japanica* | HK | LK | 5620 | kg | gill plates | T | W |
| 2019 | *Mobula tarapacana* | HK | LK | 250 | kg | fins^†^ | T | W |
| 2019 | *Mobula tarapacana* | HK | LK | 7785 | kg | gill plates | T | W |

^*^it is unclear if these were live specimens that were traded or if there was an error in the term entered.

^†^it is highly unlikely that the fins of these species were exported as that has never been documented particularly given that mobulid rays only have a small dorsal fin. It is likely that these were gill plates that were erroneously reported as fins.

^‡^missing units are due to gaps in reporting. Based on the volumes reported, they are all likely kg
