# Supplementary material for: High bycatch rates of manta and devil rays in the “small-scale” artisanal fisheries of Sri Lanka
Source: PeerJ. 2021 Sep 8;9:e11994. doi: 10.7717/peerj.11994 (PMC8434810; doi:10.7717/peerj.11994)
Supplement: Supplemental Information 3 [file peerj-09-11994-s003.docx]

**Table S2:** The global (*IUCN Red List Assessment Results, 2021)* and regional (Arabian Seas and Adjacent Waters - (Jabado et al., 2017)) IUCN Red List categories for mobulid rays.

|  | **GLOBAL ASSESSMENT** | | | **REGIONAL ASSESSMENT** | |
| --- | --- | --- | --- | --- | --- |
|  | *Status** | *Year Assessed* | *Population Trend* | *Status** | *Year Assessed* |
| *Mobula birostris* | Endangered | 2020 | Decreasing | Vulnerable | 2017 |
| *Mobula mobular* | Endangered | 2020 | Decreasing | Endangered | 2017 |
| *Mobula tarapacana* | Endangered | 2019 | Decreasing | Endangered | 2017 |
| *Mobula thurstoni* | Endangered | 2019 | Decreasing | Endangered | 2017 |
| *Mobula kuhlii* | Endangered | 2020 | Decreasing | Near Threatened | 2017 |

**Threatened IUCN Red List Categories: Critically Endangered, Endangered, and Vulnerable.*
