# Supplementary figures and images for: High bycatch rates of manta and devil rays in the “small-scale” artisanal fisheries of Sri Lanka
Source: PeerJ. 2021 Sep 8;9:e11994. doi: 10.7717/peerj.11994 (PMC8434810; doi:10.7717/peerj.11994)

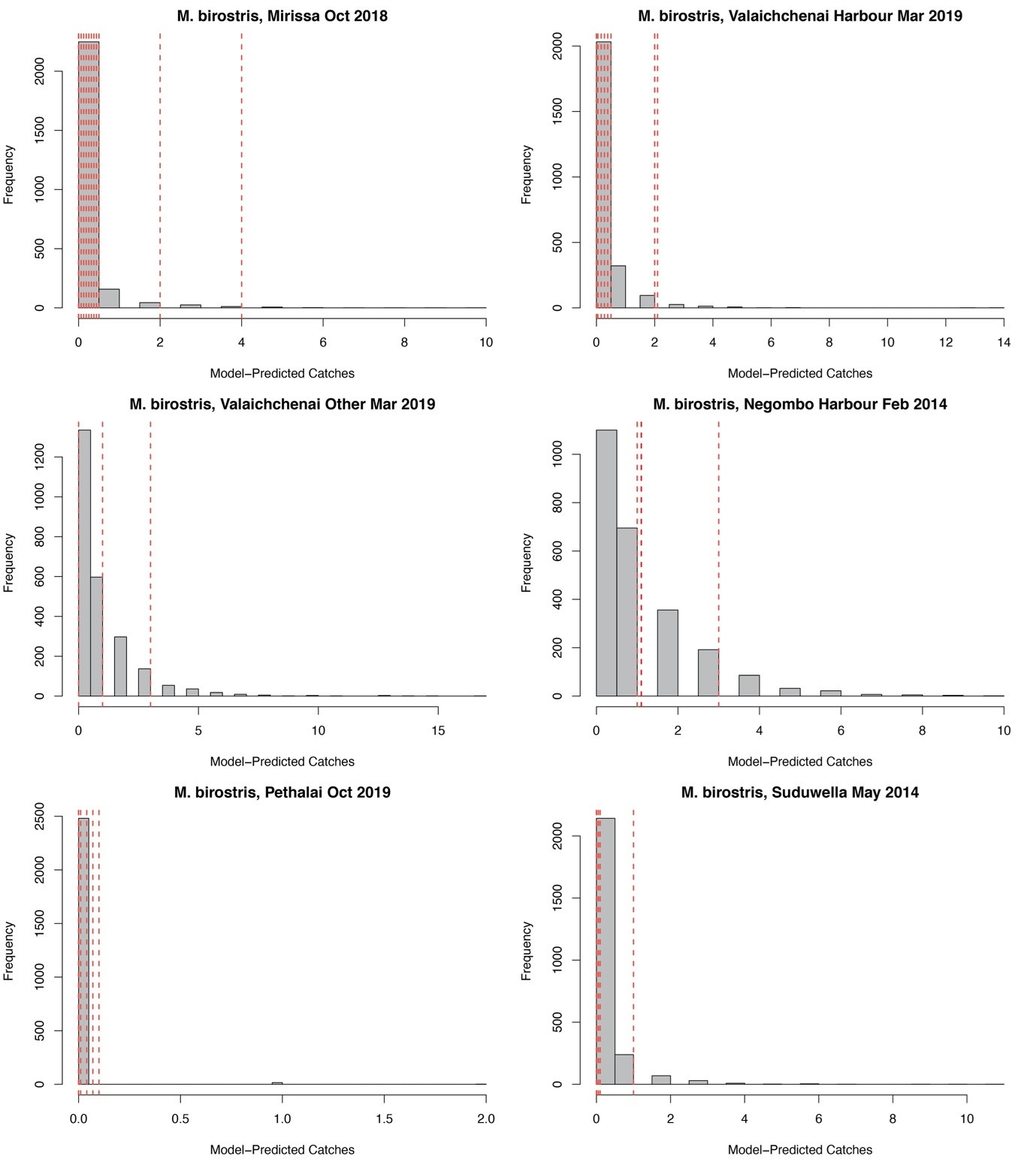

Supplement: Supplemental Information 4 — Histograms (in grey) represent the model-predicted distribution of possible catches at a given market or landing site and month. Vertical dashed lines indicate the observed landings in the respective month for each landing site. Dashed lines are jittered on the x axis for clarity. [file peerj-09-11994-s004.jpg]

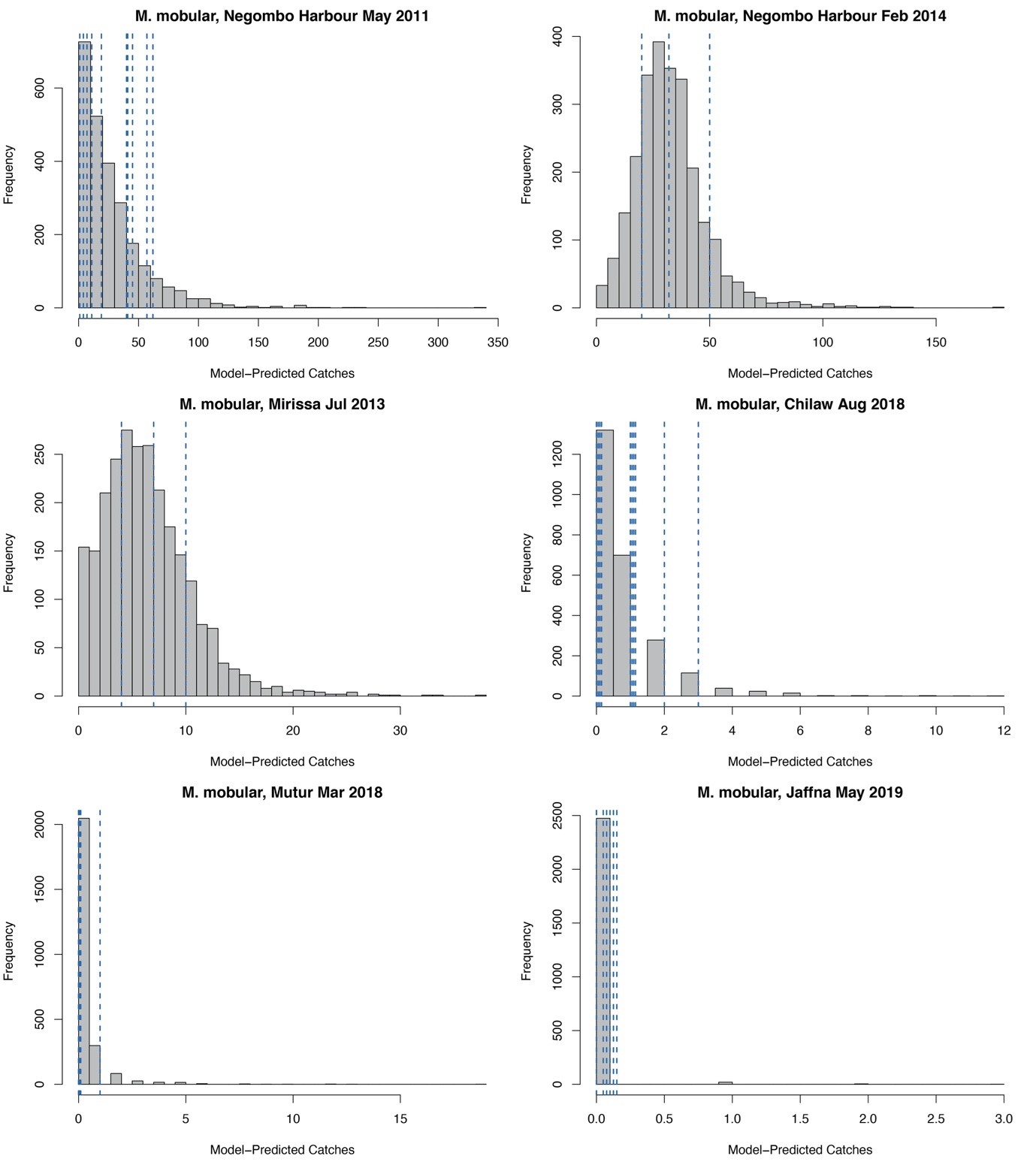

Supplement: Supplemental Information 5 — Histograms (in grey) represent the model-predicted distribution of possible catches at a given market or landing site and month. Vertical dashed lines indicate the observed landings in the respective month for each landing site. Dashed lines are jittered on the x axis for clarity. [file peerj-09-11994-s005.jpg]

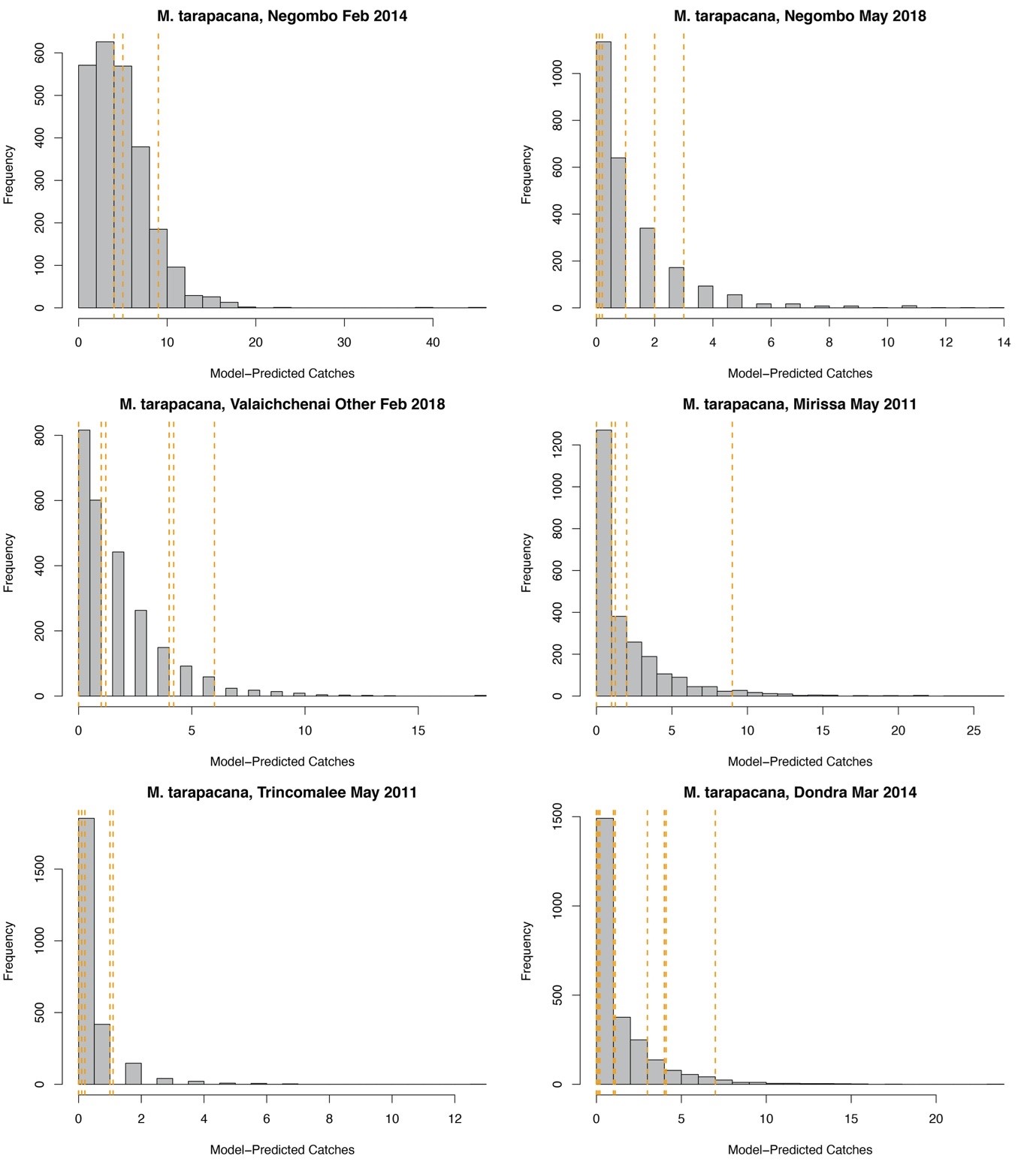

Supplement: Supplemental Information 6 — Histograms (in grey) represent the model-predicted distribution of possible catches at a given market or landing site and month. Vertical dashed lines indicate the observed landings in the respective month for each landing site. Dashed lines are jittered on the x axis for clarity. [file peerj-09-11994-s006.jpg]

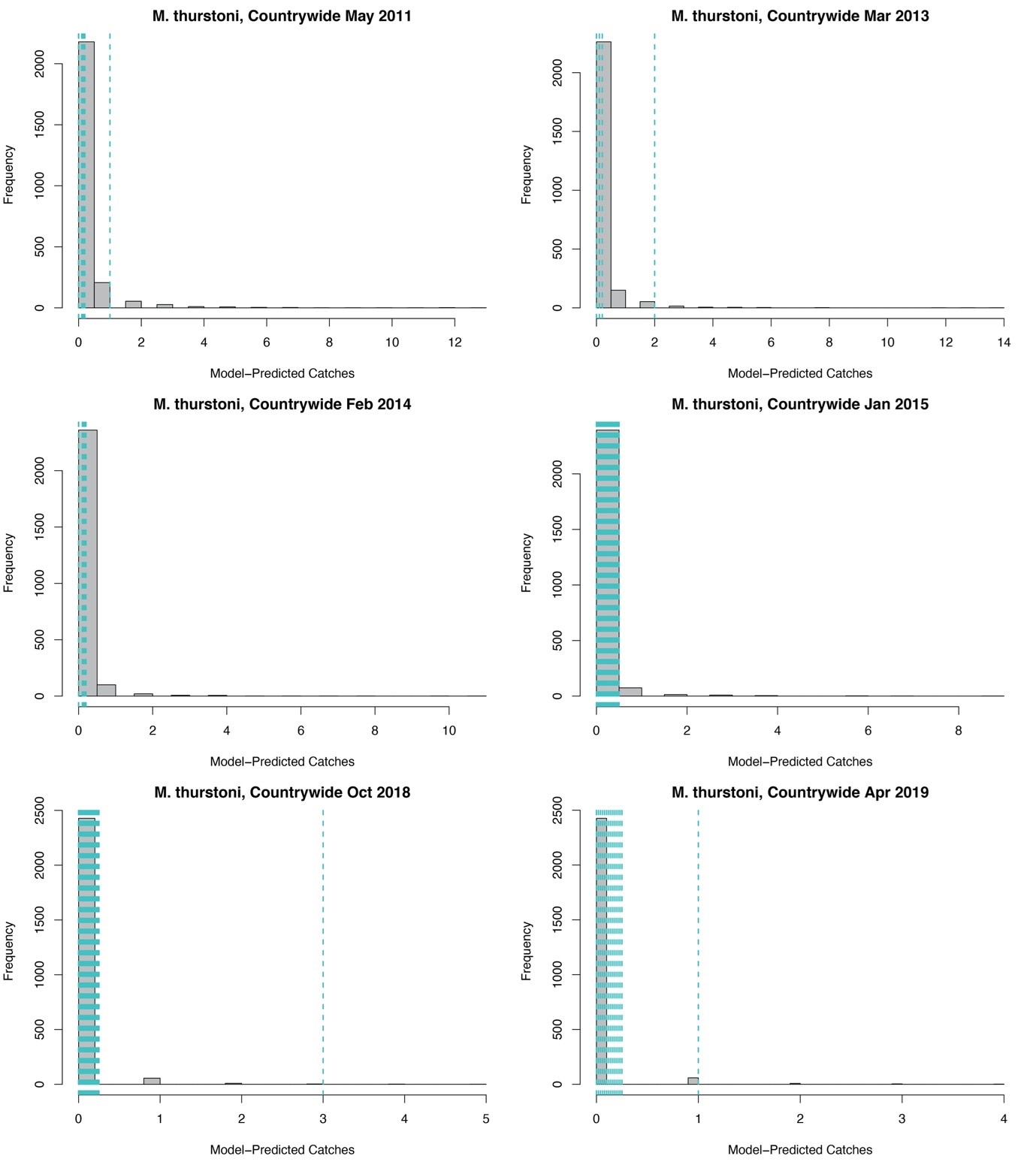

Supplement: Supplemental Information 7 — Histograms (in grey) represent the model-predicted distribution of possible catches across all markets for a given month. Vertical dashed lines indicate the observed countrywide landings in the respective month. Note that the M. thurstoni component of the state-space model did not have market-level effects, as landings were rare compared to other mobulid species, and estimating market-level effects was therefore not feasible. Dashed lines are jittered on the x axis for clarity. [file peerj-09-11994-s007.jpg]

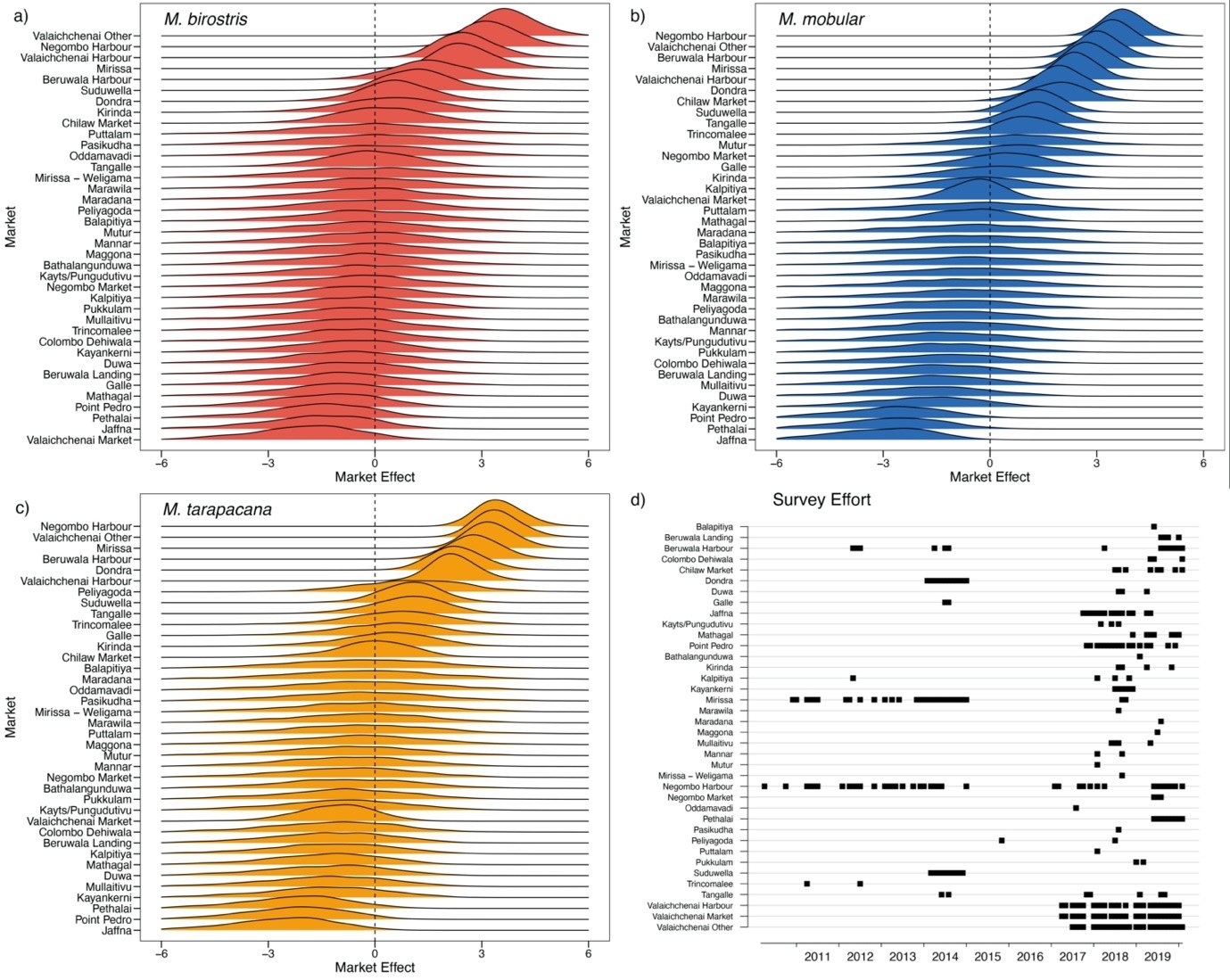

Supplement: Supplemental Information 8 — The ridges in a-c represent the posterior distributions of the mean market random effects on catch rates added (in log space) to the mean country-wide landings and then fit to market-level observed catches in each month. Note that Mobula thurstoni was not modelled with market-level random effects. (d) indicates months with survey effort (black squares) at each market. [file peerj-09-11994-s008.jpg]
